# Towards Wideband Characterization and Modeling of In-Body to On-Body Intrabody Communication Channels

**DOI:** 10.3390/bioengineering13010042

**Published:** 2025-12-30

**Authors:** Matija Roglić, Yueming Gao, Željka Lučev Vasić

**Affiliations:** 1 University of Zagreb Faculty of Electrical Engineering and Computing, 10000 Zagreb, Croatia; 2 School of Physics and Information Engineering, Fuzhou University, Fuzhou 350108, China; fzugym@163.com

**Keywords:** intrabody communication (IBC), in-body to on-body (IB2OB) communication, capacitive coupling (CC), implantable systems

## Abstract

Implantable intrabody communication (IBC) is a method that enables low-power, high-security communication between implanted in-body devices that could track biomedical signals and an on-body receiver by using the human body as a communication medium. As the human body consists of various tissues that each have different conductivity, this paper explores the effects of the conductivity of the communication medium on the channel gain over a wide frequency range from 10 MHz up to 300 MHz through the measurements and two models: an electrical circuit model and a FEM simulation model. Measurements are conducted using a liquid phantom with varying conductivity values from 0 S/m up to 1 S/m, covering most human tissues in the frequency range of interest. The circuit and FEM models are designed to mimic the measurement setup in order to verify the measurement results. Results show that the circuit model predicts the communication channel characteristics well at lower frequencies but cannot account for the influence of the measurement setup at higher frequencies. The influence of wire inductances, which can cause a resonant behavior when measuring at frequencies above 100 MHz, was observed using the FEM model. The results also show that the higher the conductivity of the tissue in which the device is implanted, the lower the gain of the signal, with the difference in gain being more prominent when capacitive termination with a high-impedance load is used instead of low-impedance termination. These findings provide valuable insight for selecting the appropriate interface (low-impedance vs. high-impedance termination) across specific frequency ranges for in-body to on-body (IB2OB) communication devices, while illustrating the effect of tissue conductivity on an IBC channel, thereby supporting the optimized design and implementation of reliable IB2OB communication systems.

## 1. Introduction

Intrabody communication (IBC) represents a method that strives to improve the efficiency and safety of wireless communication for devices that are placed on or inside the human body by using the human body itself as a communication medium [1,2]. By connecting two or more devices, a wireless body area network (WBAN) can be formed. A WBAN can then be used to monitor vital functions in the human body, to transmit measured data between the devices and the user, and to perform actions such as drug administration or electrical stimulation. Such autonomous networks could help with the aging population and relieve the burden on the medical sector [3].

To achieve efficient and secure WBANs, current standard wireless communication methods such as Bluetooth and ZigBee have proven to be inadequate due to their high energy consumption and privacy concerns [4,5,6,7]. Comparing the energy consumption per bit of the standard wireless communication methods and IBC, IBC can achieve several hundred times lower consumption at higher data rates [8]. Furthermore, standard communication methods operate at higher frequencies than IBC, and the signal can be detected up to 5 m from the source, which poses a security risk for external intruder attacks. On the other hand, the electro-quasistatic IBC operates at frequencies below 20 MHz and emits signals that can be detected up to 15 cm from the human body, which means that the privacy of the signals is drastically improved compared to standard communication methods [7]. Moreover, in 2020, and as of 2023, the FDA has issued an announcement warning against the use of Bluetooth Low Energy technology for communication with medical devices due to a cybersecurity vulnerability that would allow an unauthorized user to crash the device and render it inoperable, or access functions normally only available to the authorized user [9]. The security issue of an unauthorized user having the ability to crash the device or alter its function is especially egregious for implantable devices, where the user would not be able to manually restart or remove the malfunctioning device in case of such an attack. For these reasons, a secure and energy-efficient communication method must be developed for implantable devices.

Two main methods for achieving IBC between transmitter and receiver devices are capacitive (CC) and galvanic coupling (GC) [1,10,11,12,13,14,15], with the main difference between them being the placement of the electrodes, which affects the dominant signal return-path mechanism. In galvanic coupling, both electrodes of the transmitter and receiver devices are placed in direct contact with the body, and the signal forward and return paths are primarily confined within biological tissue. In capacitive coupling, a signal electrode (SE) is placed toward the human body, while the ground electrode (GE) is intentionally oriented or insulated from the body, so the return path is formed through parasitic capacitances between the body, the device, and the surrounding environment. This creates a forward path through the human body, and the return path is closed through the parasitic capacitances between the environment and the GE of the transmitter (Tx) and the receiver (Rx). The capacitive method allows higher achievable data rates and lower path loss compared to the galvanic IBC method, especially at longer communication distances on the body [2]. Since the capacitive coupling setup requires the GE to face the environment, it was considered unsuitable for communication in implantable systems. Nevertheless, in [16], Zhang et al. proposed a method in which the GE was insulated to ensure the GE remains floating and to achieve capacitive coupling. The electrical circuit model representing an in-body to in-body (IB2IB) communication was explained, and measurements were made using large devices with only the electrodes immersed in the water. Further papers have investigated the aforementioned method and the characteristics of the implantable intrabody communication channel [8,17,18,19,20]. In [17], Li et al. explored an in-body to an on-body (IB2OB) case by creating a simulation model and a measurement setup using a pig. However, during both simulation and measurement, the Tx device was positioned outside the body, while only the Tx electrodes were implanted inside the body. In their second work [18], Li et al. explored the IB2IB scenario using finite element modeling and real-world measurements, yet again, only the electrodes were fully implanted, while the rest of the measurement setup was placed outside the body. Moreover, using a 50 Ω termination in their measurement setup resulted in lower gain at lower frequencies, which is undesired [21].

To properly measure the properties of an implantable capacitive IBC, one or both devices must be fully implanted within the body and sealed; otherwise, additional parasitic capacitances may form outside the body, resulting in overly optimistic results [22]. In addition, the same paper by Jiang et al. argued that encapsulation of the GE was detrimental, as it may not ensure capacitive coupling but instead adds additional loss due to additional impedance in the return path. The paper, however, failed to explore a wider frequency range, opting only for a single frequency measurement at 21 MHz.

There exists a number of works conducted by various researchers that showcase the possible applications of IBC in implantable systems to transmit biomedical data or to enable communication between implanted devices: transmitting endoscopy data [23], transmitting microbiome redox sensors data [13], neural communication between a healthy nerve and an injured one [24], communication and synchronization between leadless pacemakers [25], and transmission of neural signals within the brain [26]. However, these works mostly showcase the application at a single frequency or explore a narrower frequency band than the one explored in this paper. While electromagnetic interactions with biological tissues have been extensively explored in fields such as MRI, bioimpedance measurements, and impedance tomography, these approaches primarily focus on tissue characterization and imaging rather than on establishing communication links through the body. Nonetheless, relevant insights into tissue losses and frequency-dependent behavior can be drawn from studies into MRI tissue absorption [27] and electrical impedance measurements, particularly at lower frequencies where impedance-based techniques operate [28,29].

This paper aims to expand upon the previous research by developing a real-world, measurement-based IB2OB IBC circuit model for lower frequencies, representing the communication channel in the electro-quasistatic (EQS) range, as well as developing a finite element method (FEM) simulation model to depict the higher frequencies of the communication channel. Furthermore, a measurement model using a fully implanted device to achieve a real IB2OB scenario is created and presented. This work also explores a 10–300 MHz frequency band, which is a much wider frequency range than any previous work in the field of implantable IBC communications. In addition, as the human tissues vary in conductivity values, the effect of the conductivity of the medium on the communication channel is investigated through both the circuit model and the measurement setup. Lastly, this paper compares the capacitive termination using a closed-loop buffer and 50 Ω and draws conclusions on the type of interface that should be used in an IB2OB scenario and how the implantable scenario differs from an on-body CC IBC.

## 2. Measurement Setup

**Figure 1:**
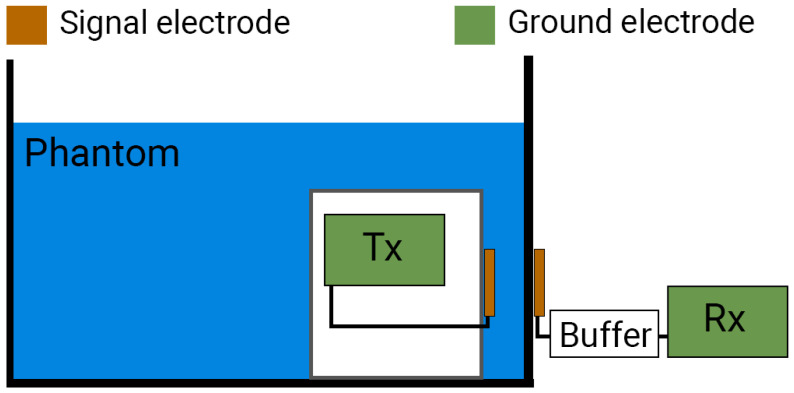
In-body to on-body measurement setup. The transmitter (Tx) is fully submerged in a liquid phantom, with only the signal electrode (SE) in contact with the phantom, while the receiver (Rx) is placed outside of the phantom. SE of Rx is placed on the box container. Both the Tx board and Rx board act as ground electrodes for their respective devices.

**Figure 2:**
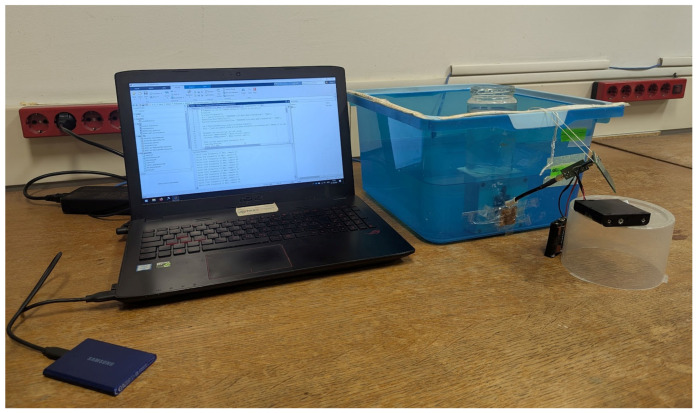
Measurement setup in the laboratory with laptop being used to transmit commands and receive data from the battery-powered devices.

For the IB2OB measurement to accurately measure the communication channel, the Tx device must be fully implanted and surrounded by the tissue. As it would be extremely invasive to perform these basic measurements on living beings, a liquid phantom is used instead to approximate the communication channel. Using a simple liquid phantom also allows exploration of the effects of conductivity, something that would not be possible in human tissue. The measurement setup consists of the following parts: a plastic box container containing a liquid phantom, battery-powered Tx and Rx, a buffer, and a laptop that serves as a control unit to send commands via a Bluetooth connection to the devices and receives and displays the measured data. The measurement setup schematic is displayed in Figure 1, while a photo of it can be seen in Figure 2.

### 2.1. Components of the Measurement Setup

#### 2.1.1. The Liquid Phantom

The liquid phantom used in the measurement setup is made using demineralized water and varying amounts of pure sodium chloride to achieve different conductivities. The demineralized water is poured into a 39 cm by 35 cm by 20.5 cm plastic container until it reaches a height of approximately 10 cm, resulting in 13.65 L of liquid phantom with 0 S/m conductivity at room temperature. The conductivity is incrementally increased in 0.1 S/m steps by adding approximately 4 g of sodium chloride at a time. After each addition, the solution is thoroughly stirred to ensure a homogeneous mixture. A handheld conductivity meter, WTW Cond 340i, is used to monitor the changes in conductivity and temperature. Once the desired phantom conductivity is achieved, a sample of the phantom is taken from the plastic container to measure the dielectric properties across the whole frequency range using a SPEAG DAK 12 probe. The measured data are then also used to calculate the components of a circuit model described in Section 3.2. In total, 11 different phantoms are created across the 0 S/m to 1 S/m conductivity range in 0.1 S/m increments.

The relative permittivity of the phantom is not altered during measurements but only monitored with the SPEAG DAK 12 probe to ensure that the value does not change significantly. The measured relative permittivity of the phantom is approximately 85 for all conductivity values. It is possible to lower the relative permittivity value of water-based liquid phantom by adding sucrose or dextrose [24,25]. However, many tissues have a relative permittivity higher than that of water, especially at lower frequencies [26]. While it is possible to achieve higher relative permittivity, in the range of 190, with saturated aqueous solutions of n-Methyl Formamide, creating these phantoms is expensive due to the cost of the ingredients [25], and the achievable relative permittivity is still below that of some tissues such as brain gray matter (319.67 at 10 MHz) and cerebellum (464.68 at 10 MHz) [26]. While there are recipes for solid phantoms that can achieve high permittivity [24,27], they are unsuitable for measurements in implantable case scenarios, as it would be extremely difficult to insert the measurement devices into such phantoms. Therefore, to study the influence of relative permittivity, new recipes should first be developed for liquid phantoms that allow for the manufacture of high-permittivity phantoms.

While the measurements on the human body would produce the most accurate results, in the case of the implantable IBC, direct in vivo measurements on humans would be extremely invasive, raise significant ethical concerns, and require substantial additional development of implantable devices to ensure full biocompatibility, miniaturization, and overall safety. For these reasons, liquid phantoms are used as a substitute for human body measurements to acquire vital information for the future development of the devices.

#### 2.1.2. Battery-Powered Transmitter and Receiver

**Figure 3:**
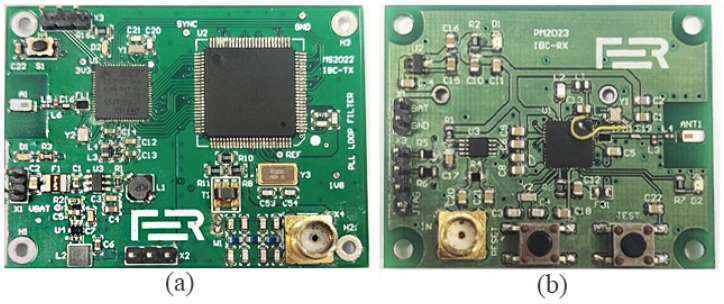
Battery-powered transmitter (**a**) and receiver (**b**) used for measurements. The detailed description of the board layout is described in [8,19].

For measuring the communication channel transmission characteristics, wearable-sized battery-powered devices shown in Figure 3 are used to achieve realistic measurements, as using large measuring devices would result in optimistic gain levels [30]. Both devices are custom-made at the University of Zagreb Faculty of Electrical Engineering and Computing.

Tx is a four-layer printed circuit board (PCB), measuring 50 mm by 55 mm by 30 mm, with components mounted on the top and bottom layers, while the middle layers are dedicated to ground and power. The entire transmitter board functions as a large GE in our measurement setup. The board includes a direct digital synthesis chip, AD9910, which can generate a sine wave signal up to 400 MHz. This signal is then output through the SMA connector to the SE. The frequency of the generated signal is set by sending a command via Bluetooth from the laptop, which serves as the measurement manager, sending commands and receiving data from the Rx, also via Bluetooth. The PCB is powered by four AAA batteries, ensuring a long operating time.

Before measurements, Tx is placed inside a non-conductive, waterproof box. The device is further fixed in place inside the box with polystyrene foam to minimize the movement of the device during measurements. A 3 cm by 3 cm copper plate is placed on top of the box and serves as an SE.

Rx is a two-layer PCB, also measuring 50 mm by 55 mm by 30 mm, with all components on the top layer. The bottom layer is almost entirely a ground layer and acts as a GE. The input sine wave signal is passed through the logarithmic amplifier AD8310, which converts it to its decibel equivalent [31]. The analog values are then converted to digital values by the integrated analog-digital converter on the STM32WB55 microcontroller and sent via Bluetooth to the laptop. The input impedance of the Rx board is 50 Ω. The Rx board is powered by four AA batteries.

#### 2.1.3. Buffer

Since the impedance seen at the receiver interface is on the order of several kiloohms [21], measurements performed with a 50 Ω termination will result in higher loss, as the voltage drop over the device will be lower compared to the rest of the communication channel [21,32]. Furthermore, in an on-body CC, due to the capacitive return path, adding a resistive termination creates a pole in the channel transfer function, resulting in a high-pass response [21]. For this reason, Maity et al. argue that a high impedance receiver with capacitive termination should be used when performing IBC measurements in voltage mode [21]. The work of Avlani et al. [33] demonstrates the use of a high-speed buffer IC, BUF602 from Texas Instruments, for on-body measurements in a wide frequency range. For measurements in this work, the same buffer setup is used to explore the effects of capacitive termination in an IB2OB case.

The buffer connects via an SMA connector to the SE, which is placed on the side of the plastic container filled with phantom, and provides a high impedance termination, while the output matches the 50 Ω input impedance of the Rx and is connected via another SMA connector to the PCB board. The input impedance of the buffer is 1 MΩ with a capacitance of 2.1 pF.

### 2.2. Measurement Procedure

The overall measurement setup will be briefly summarized in this chapter for clarity. A liquid phantom is made by mixing sodium chloride and demineralized water to achieve a specific conductivity ranging from 0 S/m to 1 S/m, covering most of the human body tissue conductivity values [34]. The Tx is enclosed within a waterproof non-conductive box and immersed in the phantom to mimic a realistic IB2OB scenario. Only the Tx SE is in contact with the phantom. The Rx is located outside the phantom, with the Rx SE placed on the side of the plastic container. When measurements are performed with a low-resistance termination of 50 Ω, no buffer is connected between the receiver SE and the Rx. In contrast, when a capacitive termination is used, a high-impedance buffer matching the body impedance and Rx input impedance is connected between the receiver SE and the Rx.

Measurements are conducted over a frequency range between 10 MHz and 300 MHz in 5 MHz increments. Each frequency point is sampled 100 times, and the mean measured power at the Rx is expressed in dBm. To determine the channel gain, the baseline is measured by shorting the Tx and Rx, and this value is subtracted from the measured power at the Rx. Measurements are conducted at a constant room temperature.

## 3. IB2OB CC IBC Models

### 3.1. EQS Circuit Model

When considering the properties of an implantable IBC communication channel, if the observed frequency range is low enough, the propagation wavelength through the system is several orders of magnitude larger than the channel lengths. Due to this, the phase of the signal remains almost constant, and magnetic fields do not contribute to the communication channel. In this scenario, we can assume the communication channel can be approximated as EQS as long as the ratio between the approximation error and the developed electric field $\vec{E}$ is less than 1 as expressed by Equations (1) and (2) [35]

(1) $$\vec{E}={\vec{E}}_{EQS}+{\vec{E}}_{error}$$

(2) $$\frac{E_{error}}{E}=\omega^{2}\mu \epsilon L^{2}\ll 1.$$

The limit of the EQS frequency *f* can then be calculated using Equation (3) for each specific human tissue material as long as the permittivity ε of the tissue is known. Since human tissue is non-magnetic, the permeability *µ* of human tissue is assumed to be equal to the permeability of the air. The size of the electrode is denoted by *L*.

(3) $$f\ll \frac{1}{2\pi L\sqrt{\mu_{material}\epsilon_{material}}}$$

**Figure 4:**
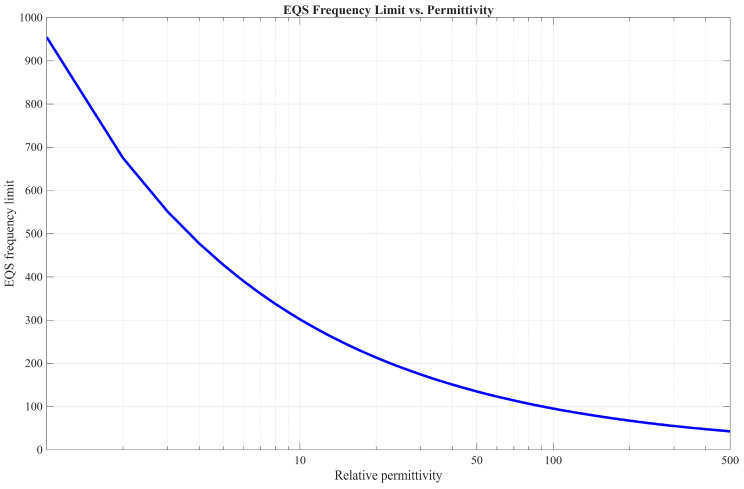
Relation between permittivity and EQS frequency limit.

The relative permittivity of human tissues differs between tissues and changes with frequency. At the low end of our measured frequency range, at 10 MHz, the highest relative permittivity is found in the cerebellum and small intestine, with values of 464.68 and 488.46, respectively, while the lowest permittivity is found in breast fat (7.89) and fat tissue (13.77) [34]. In the work of Gabriel et al. [34], it is observed that, regardless of the tissue type, permittivity decreases non-linearly with frequency, whereas conductivity increases non-linearly with frequency. The conductivity of most human tissues ranges from 0 S/m to 1 S/m for our frequencies of interest [34]. Figure 4 displays the relationship between relative permittivity and the EQS frequency limit calculated using Equation (3), where *L* equals 5 cm.

As is clear from Figure 4, higher permittivity results in a lower upper frequency limit for the EQS approximation of the communication channel. For our water-based phantom, the permittivity is approximately 85; therefore, as long as f≪105 MHz, the quasistatic near field dominates the intensity of the radiated electromagnetic fields [35], and the communication channel can be modeled with lumped elements.

### 3.2. Electrical Circuit Model

**Figure 5:**
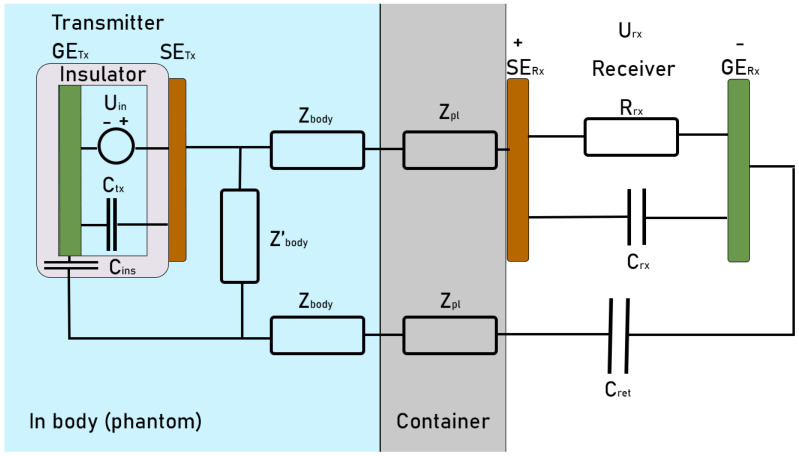
A two-layer circuit model of an IB2OB IBC communication channel.

The IB2OB IBC can be characterized as a circuit model, as shown in Figure 5. A two-layer model consisting of in-body tissue (phantom) and a container is chosen as it most accurately depicts the measurement setup. Additional layers can easily be added by replicating the first (in-body) layer in parallel to depict the most common skin–fat–muscle layer composition [36]. The tissue layer between the Tx SE and the container has a thickness of 2 cm, and its distributed impedance is represented by the complex impedances *Z*_body_ and *Z*′_body_, each modeled as a parallel RC network. The plastic container layer is represented by a single complex impedance *Z*_pl_, also modeled as a parallel RC network for the forward and return paths. However, since this layer is very thin (3 mm) and non-conductive, the parallel component is ignored as it effectively acts as an open circuit. For Tx and Rx, *C*_TX_, and *C*_RX_ represent the capacitance between the electrodes, while *R*_RX_ is the input resistance of the receiver. *C*_ret_ represents the capacitive part of the return path and couples the GE with the body, while *C*_ins_ represents the capacitance of the insulator between the tissue and the GE. Since the Tx is fully implanted in the phantom (tissue), there are no other return paths.

The impedance of the in-body layer components is calculated using Equation (4) to determine resistance based on conductivity *σ* together with the dimensions of the layer, while Equation (5) is used to calculate the capacitance from the real part of permittivity of the material ε_material_ and dimensions of the layer. In the case of *Z*_body_ and *Z*_pl_, the *d_layer_* is the thickness of each perspective layer in cases of a series component, and *A* is the surface of the electrode. For the parallel component *Z*′_body_, the length of the electrode is *d_layer_*, and *A* is the surface area of the tissue between the two SE, minus the width of the container.

(4) $$R=\frac{d_{layer}}{\sigma A}$$

(5) $$C=\epsilon_{0}\epsilon_{material}\frac{A}{d_{layer}}$$

The gain of the communication channel can then be calculated from the electrical circuit solution represented in Equations (6) and (7):

(6) $$Z_{par}=\frac{{Z\prime }_{body}(2Z_{body}+2Z_{pl}+Z_{Rx}+Z_{ret})}{{Z\prime }_{body}+2Z_{body}+2Z_{pl}+Z_{Rx}+Z_{ret}}$$

(7) $$\begin{array}{c} Gain=20\log\left|\frac{U_{Rx}}{U_{in}}\right|\\ Gain=20\log\left|\frac{Z_{par}Z_{Rx}}{\left(Z_{ins}+Z_{par})(2Z_{body}+2Z_{pl}+Z_{Rx}+Z_{ret}\right)}\right| \end{array}$$

### 3.3. Electromagnetic FEM Solver

When modeling an IB2OB IBC communication channel, changing factors such as permittivity and wavelength make it difficult to achieve realistic results using the same model across a wide frequency range. As discussed in Section 3.1, the communication channel can be accurately approximated as EQS as long as the frequency is several orders of magnitude lower than 105 MHz. To achieve an accurate representation of the communication channel at higher frequencies, an electromagnetic simulation model is created using CST Studio Suite. The model mimics the measurement setup, which consists of a plastic container with a phantom on a wooden table and Tx and Rx electrodes. All electrodes are modeled as perfect electric conductors (PEC), while the rest of the elements are assigned to the corresponding materials (plastic, wood, and liquid phantom). As the measurement setup is placed on a table, the device size is not small enough in comparison to its distance to the ground (in this case, the table), and, therefore, the distance between the table and the devices can affect the measurements [37]. For this reason, the table is also included in the simulation model.

**Figure 6:**
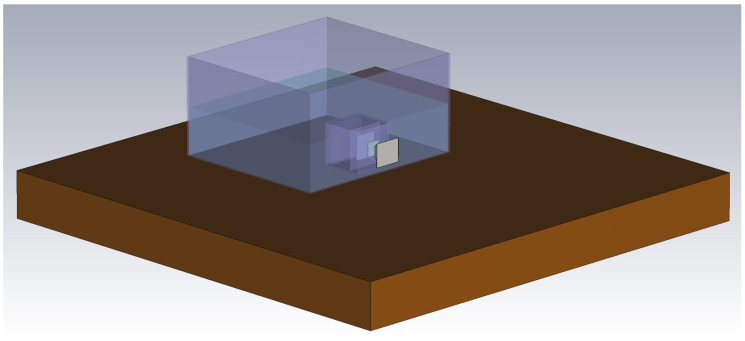
FEM simulation model in CST Studio Suite.

The boundary conditions of the model are set in CST Studio Suite as Open (add space) in all three dimensions to accurately represent an unbounded domain, as well as to ensure that electromagnetic fields decay naturally without artificial reflections. The minimum distance from the boundary to the structure was set to one-quarter of a wavelength at the center frequency of 155 MHz, while the estimated reflection level was set to 0.0001. This ensures that the outgoing electromagnetic fields are absorbed with negligible reflection and mimic the real-life measurements conducted in a laboratory environment, where the measurement setup and the table it is placed on are surrounded by air. The render of a model is depicted in Figure 6.

To achieve results, the CST Studio Suite 2024 FEM solver is used. A parameter sweep is conducted for the conductivities of the phantom tissue from 0 S/m to 1 S/m. The input impedance of the Rx is modeled as a parallel RC lumped element between the SE and the GE of the Rx.

## 4. Results and Discussion

### 4.1. EQS Characterization of an IB2OB Communication Channel

**Figure 7:**
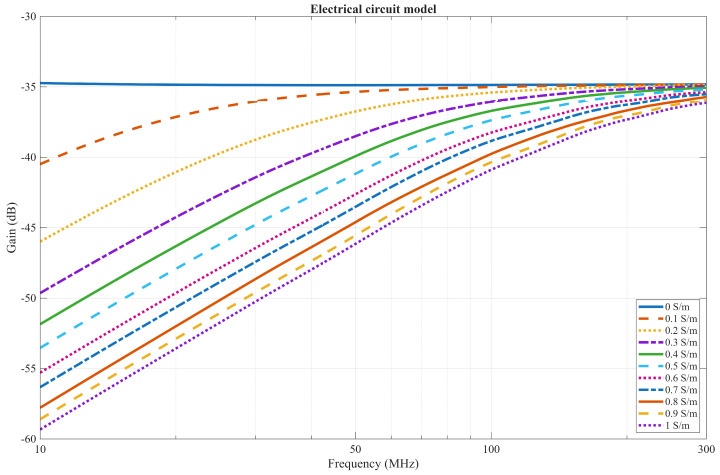
EQS model of a communication channel with a capacitive termination.

The EQS model prediction of the communication channel is displayed in Figure 7. The communication channel displays a flat band profile only when the conductivity of the phantom is 0 S/m. As conductivity increases, the communication channel profile becomes a high-pass filter, and the gain increases with conductivity.

When the conductivity of the phantom is 0 S/m, the whole system acts as an on-body CC, where the capacitive return path is formed through the air. Since the capacitor of the return path is a high impedance, Maity et al. [21] explain that using a low termination resistance at the receiver results in a high-pass response with cut-off frequency determined by the return path capacitance and termination resistance, while using the capacitive termination results in a flat-band channel response at low frequencies, which can be observed in Figure 7.

As conductivity increases, the resistive part of the impedance decreases, while the capacitive part remains the same. With the liquid phantom having a permittivity of approximately 85, the calculated capacitance *C*_body_ is about 35 pF, while the resistance *R*_body_ varies from roughly 235 Ω at a conductivity of 0.1 S/m to around 22 Ω when the conductivity reaches 1 S/m.

At low frequencies, the resistive component is more influential in the return path as long as the phantom is conductive; however, as the frequency approaches a cut-off frequency *f*_c_, the capacitive component becomes more dominant. The cut-off frequency, expressed in Equation (8) as the ratio of the material’s conductivity to its permittivity, increases with conductivity as long as permittivity remains constant.

(8) $$f_{c}=\frac{1}{2\pi RC}=\frac{\sigma_{material}}{2\pi \epsilon_{0}\epsilon_{material}}$$

**Figure 8:**
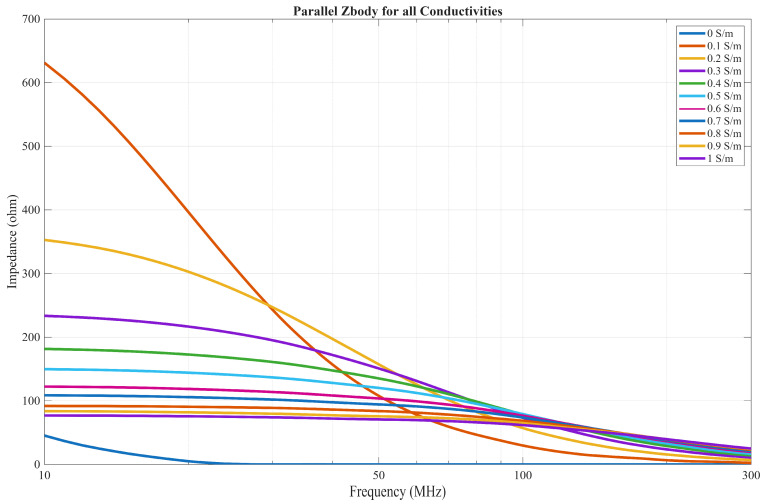
The impedance of Z′_body_ across the 10–300 MHz frequency range.

Therefore, at lower frequencies, where the resistive component dominates, a phenomenon commonly observed in GC occurs in which most of the current flows from the SE to the GE of the Tx [38]. In an IB2OB case, as conductivity increases, the resistance of the *Z*′_body_ component that is in parallel with the transmitter, as seen in Figure 8, decreases, resulting in higher current flowing through it instead of to the receiver, which results in lower gain. This effect is much less influential for an on-body CC case, as the connection between the transmitter SE and GE is only capacitive in an on-body scenario. Although a capacitive component exists between the SE and GE due to the insulation of the GE from the body, it does not ensure the capacitive coupling behavior, since it forms a common part of both forward and return current paths. Consequently, it does not influence the decreased gain caused by the rise in conductivity.

In the case where the conductivity is almost 0 S/m, the cut-off frequency is far below the measured frequency range and approaches zero, so the capacitive components dominate, resulting in a flat-band response as discussed previously, and the low impedance of *Z*′_body_ does not attenuate the signal.

### 4.2. Comparison of a FEM Solver Model with Measurement Results

**Figure 9:**
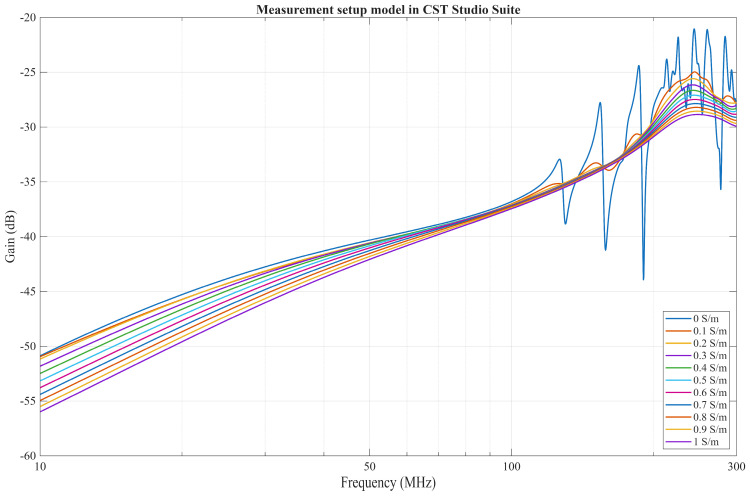
Results of Electromagnetic FEM Solver for all conductivities.

Figure 9 showcases the results of the FEM solver for all conductivity values. While the results differ from those of the EQS model when the phantom conductivity is 0 S/m, the results obtained for the other conductivity values exhibit a high-pass behavior consistent with the EQS model predictions. However, in the EQS model, the difference in gain is more prominent at lower frequencies than in the FEM solver, where the difference does exist, although it is much less pronounced. Lastly, the FEM solver showcases the resonances occurring at frequencies above 100 MHz for cases where the conductivity is low or zero, which are not captured in the EQS model.

**Figure 10:**
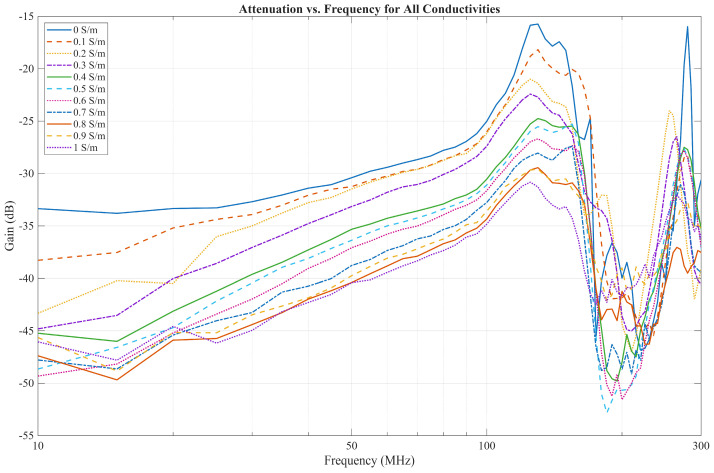
Measurement results for all conductivities across the 10–300 MHz range.

The measurement results are displayed in Figure 10. For the frequencies up to 30 MHz, the measurements align with the EQS model, as the decrease in gain is larger between lower conductivities than between higher ones. However, as the frequency increases, the measurements no longer follow the EQS model. For all conductivities, a high-pass filter behavior can be observed, similar to the FEM solver model. Gain increases by about 15 dB per decade. Around 100 MHz, the gain increases rapidly until a peak is reached around 130 MHz. After this peak, the gain sharply drops until it reaches the bottom peak around 200 MHz, then rises again in the region between 200 MHz and 300 MHz, where another peak is observed.

When comparing the FEM model solver, we see that while the FEM solver does predict the high-pass filter behavior of the IB2OB, the used simulation model does not predict the resonant behavior that occurs above 100 MHz due to its simplicity. For this reason, a more detailed model is needed to capture this phenomenon.

**Figure 11:**
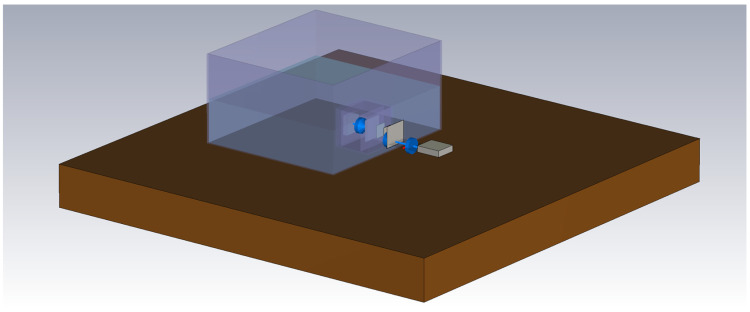
Improved simulated measurement model with battery packs and wiring represented via lumped elements (blue cones in the figure).

**Figure 12:**
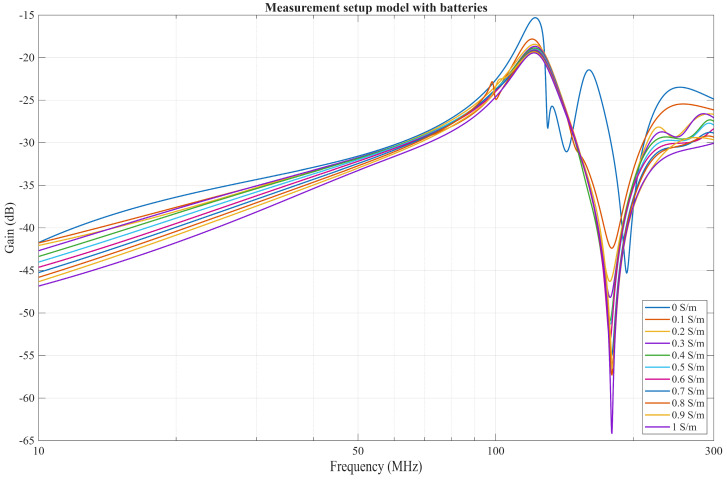
Results of improved simulated measurement model.

The improved model is represented in Figure 11 and includes an additional battery pack modeled as PEC. Their connection to the PCB board, which acts as a GE, is represented by an RL series lumped element to model the connecting wire. In the case where the Rx battery pack is placed 80 mm from the receiver, and the wire is modeled with an inductance of 400 nH and resistance of 4 mΩ (case A), resonance occurs at approximately the same frequency range observed in the measurements, as seen in Figure 12.

**Figure 13:**
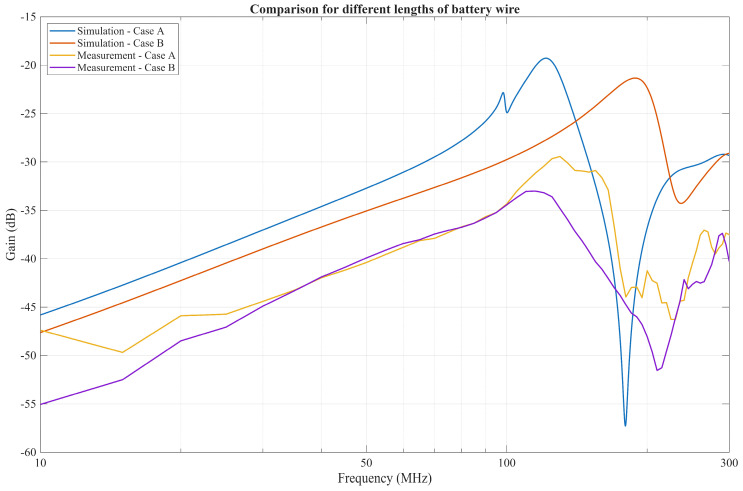
Comparison of different lengths of battery wire for two simulation cases and two measurement cases.

This resonance occurs due to the measurement setup and the inductance of the wire, which is in parallel with the parasitic capacitance between the battery pack and the PCB. While the wire can be shortened, thus lowering its inductance, this will inadvertently increase the capacitance between the battery pack and the PCB due to the reduced distance between them, resulting in comparable resonance effects. To illustrate this scenario, another simulation (case B) was performed, where the distance between the battery and the Rx PCB was set to 30 mm. The inductance and resistance of the wire in between were then linearly approximated to be around 150 nH and 1.5 mΩ. For the measurement setup, case A represents the original measurement with the battery pack separated from the RX PCB with a 47 cm long 26 AWG wire, and case B is the new measurement, where the wire has been shortened to 8 cm. The rest of the measurement setup remained the same. The results of the measurements and simulations are depicted in Figure 13.

### 4.3. Comparison of Low Resistance Termination and Capacitive Termination

**Figure 14:**
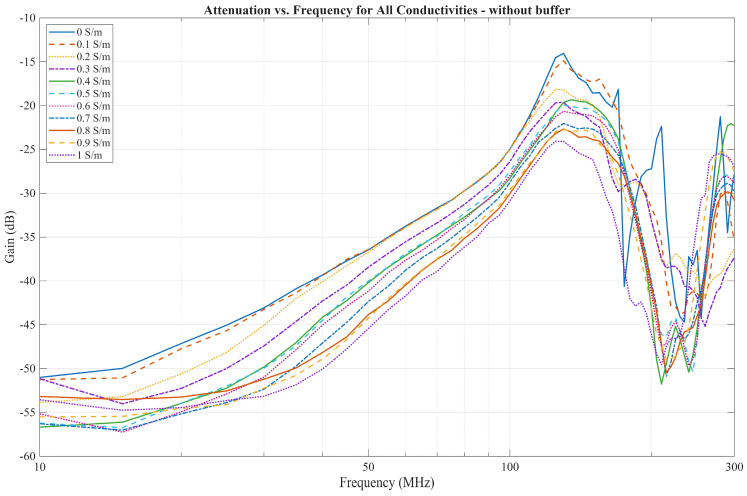
Measurement results without buffer for all conductivities across the 10–300 MHz.

In Figure 14, the measurements performed with a low input impedance of 50 Ω (without buffer) are shown. The communication channel characteristic exhibits a much sharper increase in gain from 10 MHz to 100 MHz, increasing by around 25 dB per decade when compared to the capacitive termination. The resonance effects occurring above 100 MHz are comparable to those seen in Figure 10. Despite the resonance effects, these results show qualitative similarities to those observed in previous work by Jang et al. [23].

**Figure 15:**
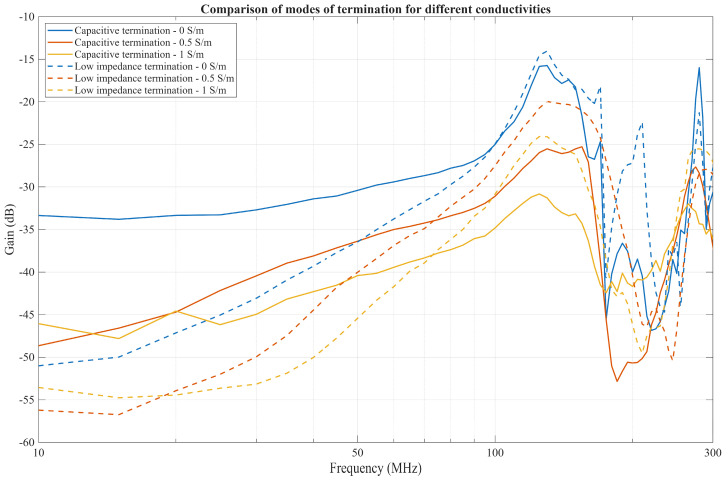
Comparison of modes of termination for conductivities 0 S/m, 0.5 S/m, and 1 S/m, where Capacitive termination is with the buffer, and low impedance without the buffer.

As seen in Figure 15, the choice of termination affects the communication channel gain in the 10 to 100 MHz frequency range for an IB2OB IBC case. At lower frequencies, gain is higher with capacitive termination. As frequency increases from 40 MHz to 60 MHz, the gain with low-impedance termination becomes higher than with capacitive termination, with the cut-off frequency shifting higher as conductivity increases. Furthermore, the difference in gain between conductivities is much less pronounced with low-impedance termination.

As discussed in [21], when measurements are performed with a low-impedance termination, the 50 Ω load results in a small voltage drop across the Rx at lower frequencies, resulting in lower gain. As frequency increases, the impedance of other components in the return path decreases (as illustrated in Figure 8), becoming comparable to the 50 Ω load. Consequently, a larger voltage drop occurs across the Rx, leading to increased gain at higher frequencies.

In addition, the difference in gain observed for varying conductivities is less pronounced with low-impedance termination. This occurs because the shunting effect through the parallel *Z*′_body_ is less dominant compared to the capacitive termination case, where the high impedance of the Rx limits current delivery and thus decreases the gain, as discussed in the previous chapter.

Lastly, it is observed that capacitive termination does not result in a flat bandpass response at lower frequencies, as it does for an on-body to on-body case for CC IBC [33], unless the tissue conductivity is 0 S/m and the entire return path is capacitive. Nonetheless, the higher gain makes the capacitive termination more appropriate for lower frequencies.

## 5. Conclusions

This paper characterizes the IB2OB IBC communication channel over a wide frequency range from 10 MHz to 300 MHz and models it using both an EQS circuit model and an FEM solver to explain the observed behavior across this frequency range. A measurement setup was developed using a liquid phantom that allows easy adjustment of conductivity within the range expected for human tissues in the observed frequency range (0–1 S/m). The effects of tissue conductivity on an IB2OB IBC channel are then explored, and a decrease in gain is observed as the conductivity increases due to the decrease in impedance of the path between the transmitter electrodes, which results in lower current flowing toward the Rx. The influence of relative permittivity was not explored in this work, as the relative permittivity was maintained at around 85. To explore the effects of permittivity, it is necessary to develop simple, inexpensive liquid phantom recipes that can simulate high-permittivity tissues, such as brain matter, at low frequencies to accurately quantify the effects of high and low permittivity scenarios on an implantable IBC communication channel. Another possible approach for future research is to use animal tissue permittivity similar to that of the human tissue, although it should be noted that the conductivity of the tissue should match the conductivity of the low-permittivity liquid phantoms to isolate the influence of permittivity on the communication channel. The tissue should also be large enough to completely encapsulate the implanted device to provide a realistic in-body scenario.

Two receiver termination modes are also compared: the capacitive termination mode and the low-impedance termination mode. The capacitive termination mode resulted in higher gain at lower frequencies when compared to the low-impedance termination. However, it did not exhibit the flat bandpass characteristic observed in other studies for an on-body to on-body CC IBC. In contrast, the low-impedance termination showed higher gain above 50 MHz and was less affected by the conductivity in terms of channel gain. Therefore, in future research, it is necessary to consider the frequency band of interest and use devices with optimal termination to ensure that there is no channel loss due to impedance mismatch between the human body and the receiver. If designing a communication device intended to cover a wide frequency range, adjustable termination is desirable to achieve maximum gain across the entire frequency band.

Lastly, the resonance at frequencies above 100 MHz was observed, and the simulation model for the FEM solver was expanded to address it. The length of the wires and the presence of the battery packs were found to influence the resonance frequency. However, it was not possible to eliminate the resonance by changing the wire length in the measurement setup, as it still resulted in the resonance occurring in a similar frequency range. For this reason, when designing battery-powered devices, it is imperative to account for parasitic inductances and capacitances arising from the device layout and to take appropriate measures to mitigate resonance effects. Furthermore, the device design should lead to the miniaturization of implantable devices to better reflect the real-world human body implant scenarios. Despite the presence of resonance effects, fully immersed battery-powered devices represent a better alternative to standard measurement instruments, such as vector network analyzers and oscilloscopes, for realistically characterizing the communication channel of IB2OB2 IBC systems.

## Data Availability

The data presented in this study are available from the corresponding author upon reasonable request.

## Abbreviations

The following abbreviations are used in this manuscript:

IBC	Intrabody communication
WBAN	Wireless body area network
CC	Capacitive coupling
GC	Galvanic coupling
SE	Signal electrode
GE	Ground electrode
Tx	Transmitter
Rx	Receiver
IB2IB	In-body to in-body
IB2OB	In-body to on-body
EQS	Electro-quasistatic
FEM	Finite element method
PCB	Printed circuit board
